# Correct use of non-indexed eGFR for drug dosing and renal drug-related problems at hospital admission

**DOI:** 10.1007/s00228-020-02953-6

**Published:** 2020-07-10

**Authors:** Sarah Seiberth, Dominik Bauer, Ulf Schönermarck, Hanna Mannell, Christian Stief, Joerg Hasford, Dorothea Strobach

**Affiliations:** 1 Hospital Pharmacy, University Hospital, LMU Munich, Marchioninistr. 15, 81377 Munich, Germany; 2 Doctoral Program Clinical Pharmacy, University Hospital, LMU Munich, Marchioninistr. 15, 81377 Munich, Germany; 3 Department of Medicine IV, University Hospital, LMU Munich, Marchioninistr. 15, 81377 Munich, Germany; 4 Department of Urology, University Hospital, LMU Munich, Marchioninistr. 15, 81377 Munich, Germany; 5 Institute of Medical Data Processing, Biometrics and Epidemiology (IBE), Faculty of Medicine, LMU Munich, Marchioninistr. 15, 81377 Munich, Germany

**Keywords:** Renal impairment, Renal risk drugs, Renal drug-related problems, Non-indexed estimated glomerular filtration rate (eGFR)

## Abstract

**Purpose:**

Two to seven percent of the German adult population has a renal impairment (RI) with an estimated glomerular filtration rate (eGFR) < 60 ml/min/1.73m^2^. This often remains unrecognized and adjustment of drug therapy is lacking. To determine renal function in clinical routine, the CKD-EPI equation is used to calculate an indexed eGFR (ml/min/1.73m^2^). For drug dosing, it has to be individualized to a non-indexed eGFR (ml/min) by the patient’s body surface area. Here, we investigated the number of patients admitted to urological wards of a teaching hospital with RI between July and December 2016. Additionally, we correctly used the eGFR_non-indexed_ for drug and dosage adjustments and to analyse the use of renal risk drugs (RRD) and renal drug-related problems (rDRP).

**Methods:**

In a retrospective observational study, urological patients with pharmacist-led medication reconciliation at hospital admission and eGFR_indexed_ (CKD-EPI) of 15–59 ml/min/1.73m^2^ were identified. Indexed eGFR (ml/min/1.73m^2^) was recalculated with body surface area to non-indexed eGFR (ml/min) for correct drug dosing. Medication at admission was reviewed for RRD and based on the eGFR_non-indexed_ for rDRP, e.g. inappropriate dose or contraindication.

**Results:**

Of 1320 screened patients, 270 (20.5%) presented with an eGFR_indexed_ of 15–59 ml/min/1.73m^2^. After readjustment, 203 (15.4%) patients had an eGFR_non-indexed_ of 15–59 ml/min. Of these, 190 (93.6%) used ≥ 1 drugs at admission with 660 of 1209 (54.7%) drugs classified as RRD. At least one rDRP was identified in 115 (60.5%) patients concerning 264 (21.8%) drugs.

**Conclusion:**

Renal impairment is a common risk factor for medication safety in urologic patients admitted to a hospital. Considerable shifts were seen in eGFR-categories when correctly calculating eGFR_non-indexed_ for drug dosing purposes. The fact that more than half of the study patients showed rDRP at hospital admission underlines the need to consider this risk factor appropriately.

**Electronic supplementary material:**

The online version of this article (10.1007/s00228-020-02953-6) contains supplementary material, which is available to authorized users.

## Introduction

Renal impairment (RI), defined as an estimated glomerular filtration rate (eGFR) < 60 ml/min/1.73m^2^ [1], is a relevant health issue in Germany. About 2–7% of the adult population are affected rising to 15–25% in patients aged over 60 years [1–3]. Unfortunately, in 72% of these patients, the RI remains unrecognized and only two-thirds of those, who are aware, are in medical care [2]. Adjustment of drug therapy is an important issue of patient safety in this patient group to avoid adverse drug reactions (ADR).

‘Renal risk drugs’ (RRD) either show altered pharmacokinetics or pharmacodynamics in RI or directly affect renal function [3, 4]. Dosage reduction and discontinuation of the drug or possibly harmful drug combinations have to be considered to avoid accumulation or nephrotoxicity, potentially leading to ADR [5, 6]. Additionally, drug activation (e.g. vitamin D) or pharmacological effectiveness (e.g. thiazides) may be influenced by RI [7, 8]. Inappropriate or missing adjustment of RRD to renal function may cause renal drug-related problems (rDRP). Many rDRP are preventable if renal function is consequently considered. However, poor awareness of pre-existing RI and incorrect use of equations for renal function is still a problem. The use of potentially inappropriate drugs and dosages is common in patients with RI [9–12], increasing the rate of ADR [13–16].

In routine clinical practice, renal function is mostly determined using the endogenous filtration marker creatinine to calculate an eGFR or an estimated creatinine clearance (Fig. S1) [9].

To stage chronic kidney disease (CKD) for diagnosis, prognosis and treatment, the Kidney Disease Improving Global Outcomes Initiative (KDIGO) recommends the calculation of eGFR using the CKD Epidemiology Collaboration (CKD-EPI) equation and graduates eGFR in six categories [1, 17, 18]. CKD-EPI-equation estimates GFR_indexed_ for a standard body surface area (BSA) of 1.73 m^2^ and can be used to compare renal function regardless of the individual’s size and weight. Importantly, for drug dosing, the GFR_indexed_ should be individualized for every patient to the units ml/min by adjusting for the BSA calculated from actual weight and height (eGFR_non-indexed_) [1, 18, 19]. This is of special importance for patients whose BSA differs significantly from 1.73 m^2^ and for drugs with a narrow therapeutic index, yet since eGFR_indexed_ is calculated automatically by many clinical laboratories, physicians tend to incorrectly use this parameter for drug dosing.

In comparison, the Cockcroft-Gault (CG) equation considers patients’ weight and estimates a creatinine clearance in ml/min [20]. It can directly be used for drug dosing and has been the standard for dose recommendations in most summary of product characteristics (SPC) in the last decades, but it has to be discussed which weight (e.g. total, ideal, adjusted or lean body weight) to use [21–24].

In addition to CKD, acute kidney injury (AKI) has to be considered when drugs are prescribed. This is especially important in urologic patients, who often present with urinary flow obstructions at hospital admission. This may be transitory, but still overdosage and inappropriate drug selection are of immediate concern. Estimating renal function in AKI is challenging since creatinine is not in a steady-state [1].

With hospital admission, the physician on ward becomes responsible for a patient’s drug therapy. The ambulant medications are generally continued during hospital stay. Therefore, dosing errors that have been introduced in ambulant care may be continued during the hospital stay and cause ADR, if not identified and corrected at the time of hospital admission. In our hospital, a pharmacist-led medication reconciliation is supporting this process. Since RI often remains unrecognized in outpatients, screening for decreased eGFR and rDRP at this transition of care is important.

To our knowledge, no data exist about the prevalence of impaired renal function in patients admitted to an urologic department of a tertiary teaching hospital. It is currently unknown, if and which RRD are taken by these patients and if rDRP exist. Additionally, the impact of using the eGFR_non-indexed_ (actual BSA) rather than the eGFR_indexed_ (standard-BSA) in clinical routine has not been addressed yet.

The objective of this study was to determine the number of patients admitted to urological wards presenting with RI and to evaluate how many of these patients changed eGFR-categories when referring to indexed vs. non-indexed eGFR, estimated with the CKD-EPI-equation. In addition, patients with eGFR_non-indexed_ 15–59 ml/min were further analysed regarding RRD and rDRP concerning pre-existing drug therapy.

## Methods

### Patients, setting and design

We conducted a retrospective observational study of patients at the time of hospital admission to two urological wards of the University Hospital Munich, Germany, between July 2016 and December 2016. Patients were included if they were ≥ 18 years of age and received a pharmacist-led medication reconciliation at admission, generating a structured medication plan of all prescribed and over the counter drugs used. Readmissions were included, since the study was designed to represent a real-life setting and patient’s renal function may change over time.

The following data were collected: age, gender, height, weight, eGFR_indexed_, drugs at admission including drugs on-demand and scheduled medications and comorbidities known to affect renal function. Body mass index (BMI), BSA (Mosteller equation) and eGFR_non-indexed_ were calculated [18, 25, 26].

### Ethical approval

The study was conducted in accordance with the Declaration of Helsinki. Ethical approval was obtained from the ethics committee at Ludwig-Maximilians-University Munich, registration number 778-16.

### Data collection

Data were collected from the hospital’s electronical patient information system (SAP-i.s.h.med, Cerner Corporation, North Kansas City, USA) that includes medical reports, diagnoses and administrative documents. Drugs and dosages were extracted from the medication plans generated by a pharmacist at admission. Comorbidities were recorded either from SAP-i.s.h.med or derived from the indication of drugs taken, e.g. statins for hypercholesterolemia.

### Renal impairment

The eGFR_indexed_ was automatically calculated by the clinical laboratory using the CKD-EPI-equation with standardized serum creatinine [27–29]. The pharmacist estimated BSA and readjusted the eGFR_indexed_ to eGFR_non-indexed_ for drug dosing recommendations by using the equation eGFR_non-indexed_ = eGFR_indexed_/1.73 m^2^ × BSA [18].

### Identification of ‘renal risk drugs’ and renal drug-related problems

Furthermore, patients with eGFR_non-indexed_ 15–59 ml/min were identified and their medication at admission was screened for renal risk drugs (RRD) and renal drug-related problems (rDRP). RRD/rDRP were analysed for subgroups of eGFR-categories (15–29, 30–44 and 45–59 ml/min). The medications of patients with eGFR_non-indexed_ > 60 ml/min usually do not need dose or drug adaption and patients with eGFR_non-indexed_ < 15 ml/min are routinely under the care of a nephrologist; thus, they were not further analysed in this study.

The evaluation whether a drug was a RRD and whether the dosage was incorrect or contraindicated with the individual’s eGFR_non-indexed_ was based on the information given in the German SPC or the drug information database AiDKlinik® that refers to the renal dose recommendation portal Dosing® (www.dosing.de). In case of discrepancies, an additional source was consulted [30].

### Classification of renal drug-related problems

**Fig. 1:**
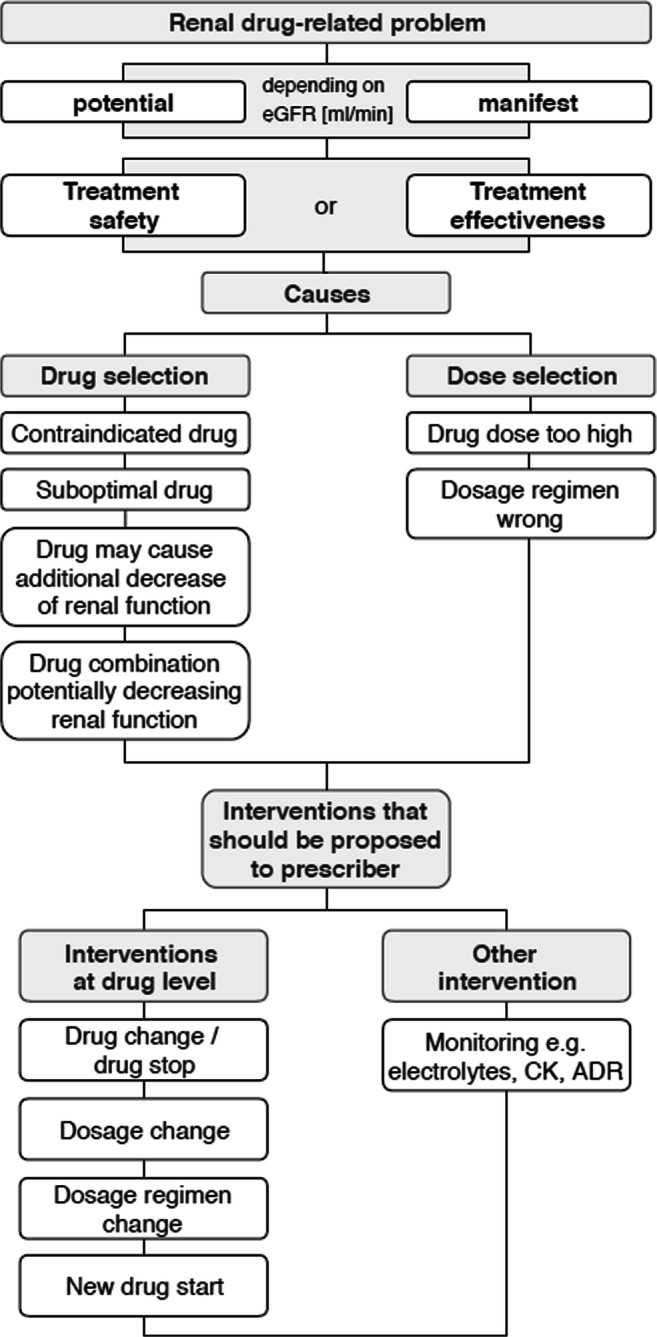
Classification of potential/manifest renal drug-related problems depending on an individual’s eGFR (ml/min). The renal drug-related problem (rDRP) of either treatment safety or treatment effectiveness is categorized in one main cause and more than one intervention might be necessary to solve rDRP. CK, creatine kinase; ADR, adverse drug reaction

The renal drug-related problems (rDRP) were classified in consensus decision by three clinical pharmacists. rDRP were categorized with regard to ‘PCNE Classification’ and previous studies concerning rDRP (Fig. 1) [3, 4, 31]. All rDRP were classified as potential or manifest depending on the individual’s eGFR_non-indexed_. Manifest rDRP are present with the current eGFR_non-indexed_. Potential rDRP concern eGFR_non-indexed_-values at threshold range (until +15 ml/min) and action must be taken when renal function decreases further. The actual incidence of adverse clinical events resulting from rDRP was not investigated.

The rDRP concern either treatment safety or treatment effectiveness and are categorized in one main cause inducing the rDRP. One rDRP may lead to one or more interventions.

The suggested interventions were related to drug level and/or need for ‘monitoring’. Monitoring refers to control of serum blood levels (e.g. electrolytes, creatine kinase) or ADR. Monitoring of serum creatinine and eGFR was not included since it is mandatory in this patient population.

### Statistical analysis

Data at admission were analysed using descriptive statistics. Qualitative variables are presented with their frequency distribution. Quantitative variables are expressed as the median and interquartile range (data without normal distribution). For comparison of the patient’s characteristics concerning age and gender, Chi-square test was used for categorical variables (independent samples) and Mann-Whitney *U* test for continuous variables (without normal distribution, independent samples). Statistical significance was accepted as *p* < 0.05.

Statistical analyses and figures were performed with Microsoft Excel® 2016 (Seattle, WA, USA) and IBM SPSS Statistics® version 25.0 (Armonk, NY, USA).

## Results

### Identification and categories of renal impairment: the impact of eGFR_indexed_ versus eGFR_non-indexed_

During the 6-month study period, 1341 patients were admitted. For 1320 (98%) patients, pharmacist-led medication reconciliation was performed. The majority of all patients were male (82.6%) and the median age 67 (18–94) years. Baseline characteristics of all patients are shown in Table 1.

**Table 1:** Baseline characteristics of all patients with pharmacist-led medication reconciliation, and for the eGFR_non-indexed_-category sub-groups. Data are quoted as the median (interquartile range) or *n* (%)

eGFR categories (ml/min)	Overall	< 15	15–59	≥ 60
No. of patients (n)	1320*	15	203	1102
Males	82.6%	66.7%	73.4%	84.5%
Age (years)	67 (18–94)	72 (33–93)	76 (30–94)	66 (18–94)
18–39	7.5%	46.7%	0.5%	8.8%
40–59	21.4%	20.0%	7.4%	24.0%
60–64	12.5%	0.0%	9.4%	13.2%
65–74	29.8%	26.7%	25.1%	30.8%
≥ 75	28.8%	46.7%	57.6%	23.2%
eGFR_non-indexed_ (ml/min)	94 (9–213)	11 (9–14)	45 (15–59)	100 (60–213)
Weight (kg)	80 (32–161)	72 (45–104)	74 (35–127)	81 (32–161)
Height (cm)	175 (148–199)	170 (156–183)	171 (148–188)	176 (150–199)
BMI (kg/m^2^)	25.8 (13.0–51.9)	23.1 (17.4–37.3)	24.6 (13.0–38.7)	26.0 (13.7–51.9)
< 18,5	2.2%	14.3%	3.0%	1.8%
18.5–24.9	38.8%	57.1%	50.5%	36.4%
25–29.9	39.8%	14.3%	33.2%	41.4%
≥ 30	19.2%	14.3%	13.4%	20.3%
BSA (m^2^)	1.97 (1.17–2.86)	1.87 (1.40–2.20)	1.88 (1.26–2.55)	1.99 (1.17–2.86)
No. of drugs at admission (n)	3 (0–20)	8 (1–13)	5 (0–17)	3 (0–17)
0	17.7%	0.0%	6.4%	20.0%
1 or 2	23.9%	6.7%	17.2%	25.4%
3 or 4	18.5%	6.7%	22.2%	18.0%
5 or 6	14.8%	26.7%	12.8%	15.1%
7 or 8	11.7%	13.3%	13.8%	11.3%
9 or 10	7.0%	20.0%	14.8%	5.4%
> 10	6.4%	26.7%	12.8%	4.9%
Comorbidities	*n* (%)	*n* (%)	*n* (%)	*n* (%)
Arterial Hypertension	655 (49.6)	13 (86.7)	140 (69.0)	502 (45.6)
Diabetes mellitus type 2	192 (14.5)	3 (20.0)	34 (16.7)	155 (14.1)
Hypercholesterolemia	317 (24.0)	7 (46.7)	62 (30.5)	248 (22.5)
Cardiovascular disease	114 (8.6)	3 (20.0)	33 (16.3)	78 (7.1)
Heart failure	38 (2.9)	2 (13.3)	18 (8.9)	18 (1.6)
Pulmonary disease	94 (7.1)	1 (6.7)	21 (10.3)	72 (6.5)
Hyperuricemia	132 (10.0)	4 (26.7)	35 (17.2)	93 (8.4)
Prostatic hypertrophy	317(29.1**)	3 (30.0**)	49 (32.9**)	265 (28.5**)
Outflow problems or obstruction of urinary tract	203 (15.4)	6 (40.0)	39 (19.2)	158 (14.3)
Hydronephrosis	250 (18.9)	8 (53.3)	87 (42.9)	155 (14.1)
Kidney transplant	6 (0.5)	1 (6.7)	2 (1.0)	3 (0.3)

*222 (16.8%) readmissions**referring to males

**Fig. 2:**
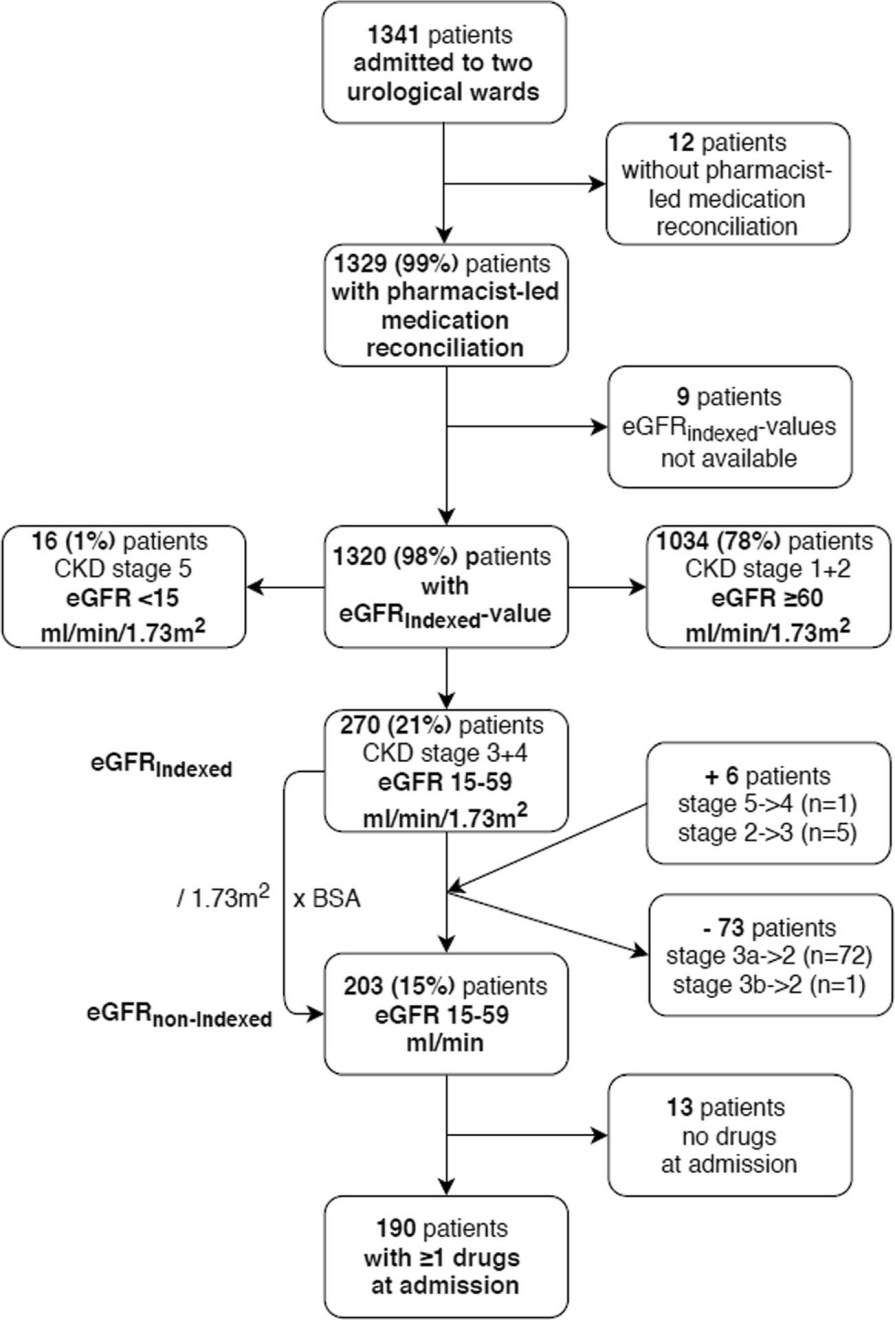
Patient flow of all patients admitted to two urological wards during 6 months

**Fig. 3:**
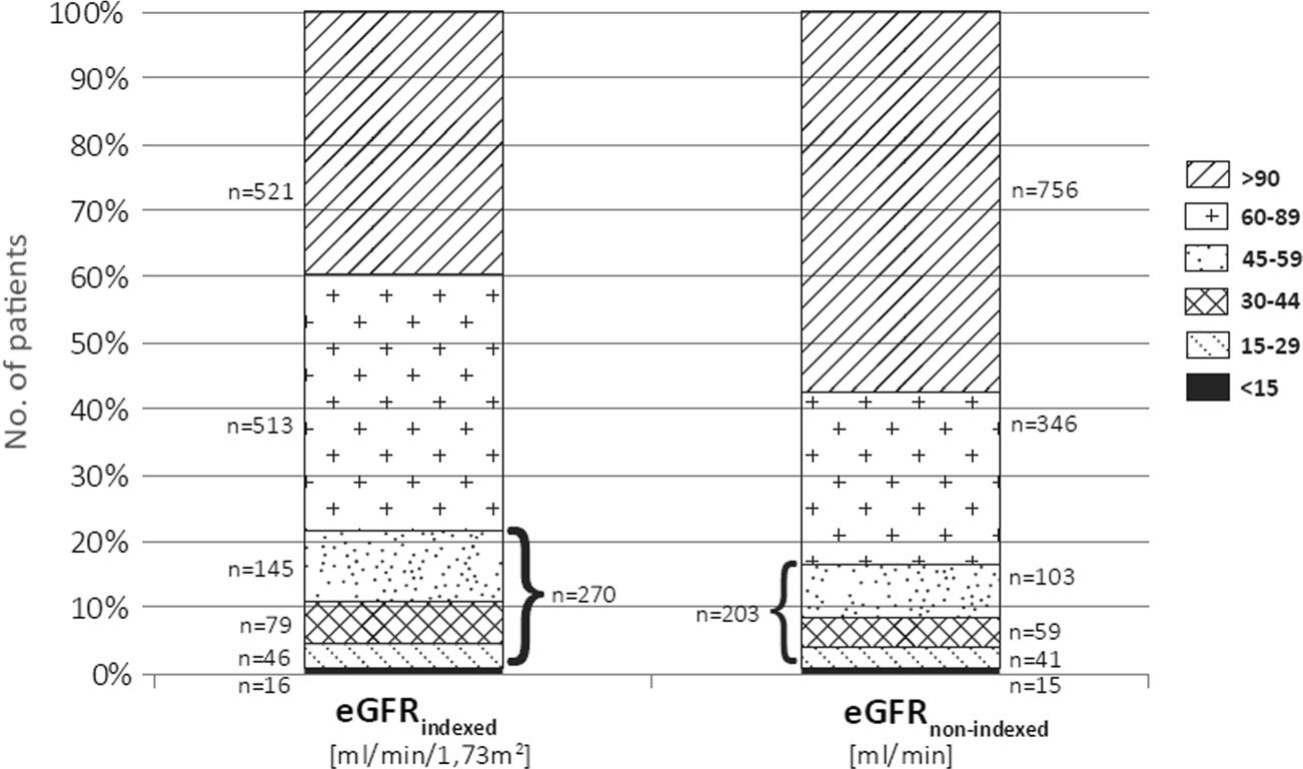
Distribution of patients with eGFR_indexed_ and eGFR_non-indexed_ according to eGFR-categories (*n* = 1320)

As there is an uncertainty regarding which formula is routinely used for drug dosing purposes in clinical routine, we investigated the impact on the number of patients with renal impairment (RI) using eGFR_indexed_ versus eGFR_non-indexed_. Two hundred seventy (20.5%) patients had an eGFR_indexed_ of 15–59 ml/min/1.73m^2^ and 16 (1.2%) patients presented with eGFR_indexed_ < 15 ml/min/1.73m^2^. However, the median BSA for all patients was noticeably higher than 1.73 m^2^ (median 1.97 m^2^, 1.17–2.86). When taking the patients’ actual BSA into account and recalculating to eGFR_non-indexed_, 67 (5.1%) patients were no longer in the critical range of 15–59 ml/min (Figs. 2 and 3).

The remaining 203 (15.4%) patients in the critical eGFR_non-indexed_-range of 15–59 ml/min were older (median age 76 (30–94) years) in comparison with patients with eGFR ≥ 60 ml/min and significantly more often presented with comorbidities such as arterial hypertension, cardiovascular disease or hyercholesterolemia. There was no significant difference in the occurrence of prostatic hyperthrophy (*p* = 0.98), diabetes mellitus type 2 (*p* = 0.32) and urinary flow obstructions (*p* = 0.08).

Comparing male and female patients with eGFR_non-indexed_ 15–59 ml/min, male patients presented with higher BMI and BSA, but no significant difference in age (*p* = 0.31), number of drugs (*p* = 0.62), and eGFR_non-indexed_ (*p* = 0.69) was found. The comorbidities differed regarding hyperuricemia and cardiovascular diseases.

Comparing patients aged < 65 and ≥ 65 years, eGFR_non-indexed_ did not significantly differ (*p* = 0.88), while older patients took more drugs (*p* < 0.05).

### RRD and rDRP in patients with eGFR_non-indexed_ 15–59 ml/min

As the eGFR_non-indexed_ should correctly be used for dosing drugs, we next deciphered the occurrence of RRD and rDRP in the patients with eGFR_non-indexed_ of 15–59 ml/min. One hundred ninety (93.6%) of the 203 patients took one or more drugs at admission (Table 2). In total, 1209 drugs were documented for these patients with a median number of 6 (1–18) drugs per patient. Out of these, 660 (54.7%) were identified as RRD with a median of 3 (0–11) RRD per patient. The 1209 drugs taken represented 218 different substances according to the Anatomical Therapeutic Chemical (ATC) classification system with 113 (51.8%) matching the criteria of RRD.

**Table 2:** Renal risk drugs (RRD) and renal drug-related problems (rDRP) in patients with eGFR_non-indexed_ 15–59 ml/min and ≥ 1 drug (*n* = 190). Data are quoted as the median (interquartile range) or *n* (%)

			eGFR categories		
eGFR_non-indexed_ (ml/min)		Overall 15–59	15–29	30–44	45–59
No. of patients	*n*	190	39	56	95
Drugs at admission	*n*	1209	278	361	570
	median (range)	6 (1–18)	6 (1–18)	6 (1–17)	5 (1–14)
Renal Risk Drugs (RRD)	*n* (%)	660 (54.7)	160 (57.5)	187 (51.8)	313 (54.9)
No. of RRD per patient	median (range)	3 (0–11)	4 (0–11)	3 (0–10)	3 (0–9)
RRD with rDRP	*n* (%)	264* (21.8**)	107* (38.5**)	85* (23.5**)	72* (12.6**)
Patients with rDRP	*n* (%)	115 (60.5)	31 (79.5)	35 (62.5)	49 (51.6)
No. of rDRP per patient	median (range)	2 (0–10)	2 (0–10)	2 (0–7)	1 (0–4)
No. of rDRP	*n*	260	105	85	70
Potential^a^	*n* (%)	108 (41.5)	14 (13.3)	61 (71.8)	33 (47.1)
with only monitoring^b^	*n* (%)	10 (9.3^#^)	0 (0.0)	6 (10.0^#^)	4 (12.1^#^)
Manifest^c^	*n* (%)	152 (58.5)	91 (86.7)	24 (28.2)	37 (52.9)
with only monitoring^b^	*n* (%)	57 (37.2^#^)	26 (28.6^#^)	14 (56.0^#^)	17 (45.9^#^)

*Drugs were counted separately, when there was a drug interaction potentially decreasing renal function (two or three drugs per interaction)

**Percentage value refers to drugs at admission

#Percentage value refers to potential or manifest rDRP

a: eGFR must be monitored, if it decreases, action must be taken

b: Monitoring as only intervention: Serum blood value (e.g. electrolytes) or adverse drug reaction must be monitored

c: rDRP is currently present with the current eGFR

Of the 660 RRD, 264 RRD led to rDRP concerning 76 different substances. This represents 21.8% of all medications and 40.0% of the RRD. One hundred fifteen patients of 190 (60.5%) had rDRP already at hospital admission. The 260 documented rDRP represented a median number of 2 (0–10) rDRP per patient (Table 2). In two cases, the rDRP was a ‘drug combination potentially decreasing renal function’ triggered by three drugs. Thus, the number of RRD (*n* = 264) is higher than the number of rDRP (*n* = 260).

### Detailed analysis of rDRP

**Fig. 4:**
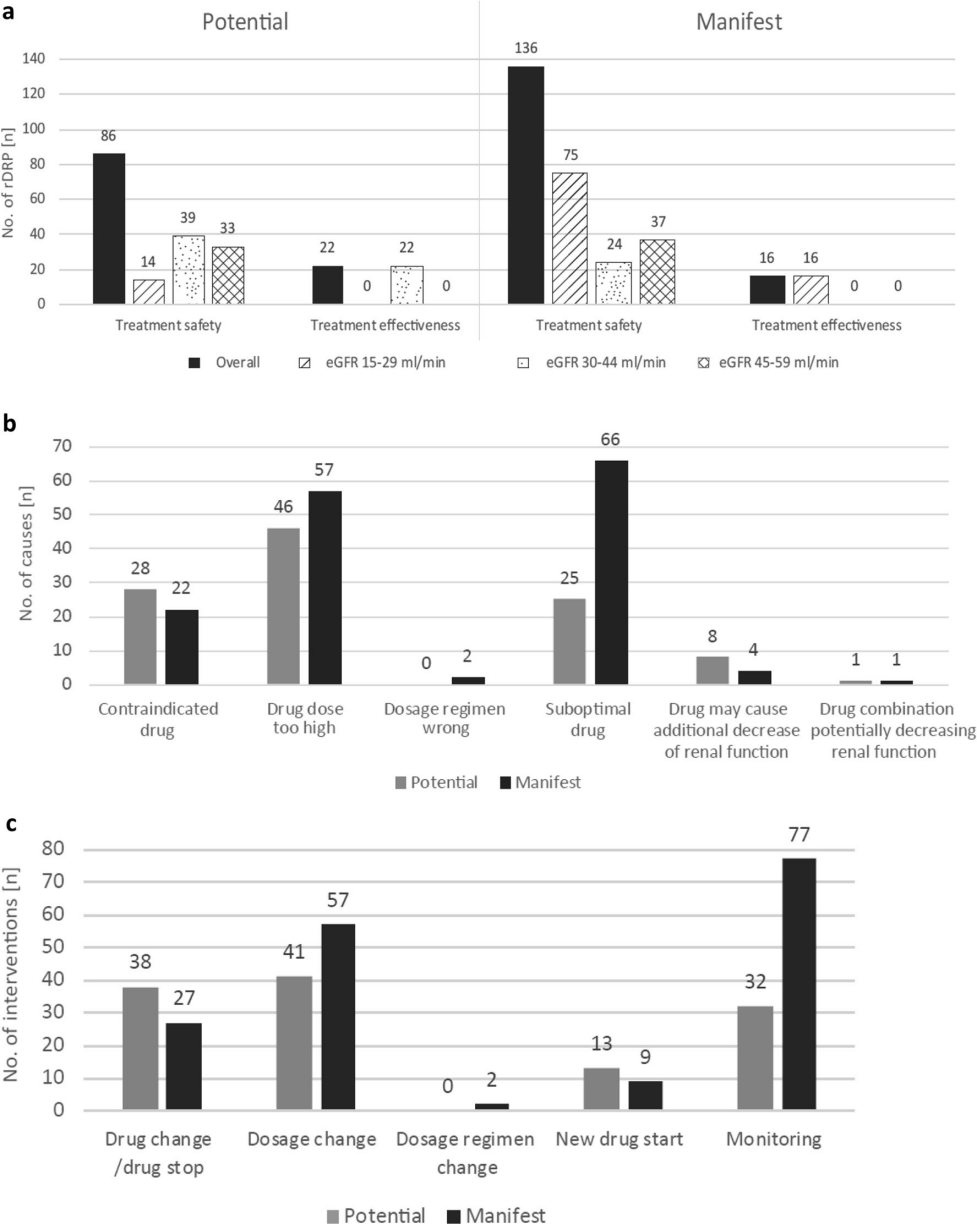
Potential and manifest renal drug-related problems (rDRP) (*n* = 260) in patients with eGFR_non-indexed_ of 15–59 ml/min and ≥ 1 drug (*n* = 190). **a** Type of rDRP. **b** Causes of rDRP. **c** Interventions that should be proposed to prescriber to solve rDRP. Potential: eGFR must be monitored, if it decreases, action must be taken. Manifest: rDRP is currently present.

For all patients with rDRP (eGFR_non-indexed_ 15–59 ml/min; *n* = 115), more manifest (58.5%) than potential (41.5%) rDRP were found (Fig. 4). Enoxaparin and colecalciferol, followed by ramipril, spironolactone, hydrochlorothiazide, simvastatin and metformin are the drugs most often associated with rDRP (Fig. S2). The most frequent intervention for manifest rDRP would have been ‘monitoring’ and ‘dosage change’, and for potential rDRP ‘dosage change’ and ‘drug change/drug stop’.

## Discussion

In this study, for the first time, the prevalence and the nature of RRD and rDRP were assessed in patients presenting with RI at admission to urological wards in a tertiary teaching hospital. Of note, we found that over half of the drugs taken at admission are RRD with 40.0% of these leading to rDRP. The use of indexed versus non-indexed eGFR has a noticeable impact on the determination of renal function, in turn influencing drug dosing and thus patient safety.

We found 21.7% of patients to have RI with an eGFR_indexed_ < 60 ml/min/1.73m^2^ at hospital admission. The number of affected patients was expected to be higher in urology, since urinary flow obstructions and prostatic hypertrophy are typical for patients admitted to urologic wards, but this is within the similar range reported by other studies for all hospitalized patients or patients admitted to internal medicine [3, 6]. There was however no difference in the occurrence of prostatic hypertrophy or urinary flow obstructions in the different eGFR-subgroups. We could not distinguish the percentage of patients with AKI or CKD or a combination of both at hospital admission, since eGFR-values from the past were rarely available.

Screening for RI at hospital admission is especially important considering that up to 72% of non-hospitalized CKD patients are not aware of their kidney insufficiency [2] and RI is an established risk factor for DRP [32]. Regarding the adjustment of drug therapy to renal function, it is vital to understand which estimation of GFR or renal clearance to use. For the classification of severity of the disease, international guidelines mainly recommend the use of the CKD-EPI-equation standardized to BSA of 1.73 m^2^ [1]. For drug dosing purposes, a measurement for renal function in ml/min (eGFR_non-indexed_ or eKreaCl) should be used [19]. Noteworthy, most patients in this study had a higher BSA (median 1.97 m^2^, range 1.17–2.86) than the standard BSA of 1.73 m^2^, which influences the calculation of eGFR_non-indexed_. Indeed, we detected a considerable shift of patients between eGFR-subgroups when readjusting for actual BSA, resulting in a distinct decrease in the number of patients to consider for drug therapy adjustment. In fact, obesity is a rising problem in the last years and has to be considered for the estimation of renal function and drug dosing. Recent reviews summarize that for drug dosing, eGFR_indexed_ underestimates renal function, and therefore, eGFR_non-indexed_ should be used [22, 23]. Indeed, our data suggest that the patient’s individual weight and height have to be increasingly considered, and subsequently, eGFR_indexed_ must be adjusted to patients’ actual BSA for correct drug dosing. This recalculation to eGFR_non-indexed_ for drug dosing was not considered in comparable studies [3, 4]. Nevertheless, in our opinion, this is a crucial point to be stressed, since many practitioners are not aware of the differences between the calculations and their impact on determining renal function and thus drug dosing.

This study focused on patients with an eGFR_non-indexed_ 15–59 ml/min for two reasons. Firstly, adjustment of drug therapy is usually necessary for GFR_non-indexed_ < 60 ml/min [30]. Secondly, whereas patients with < 15 ml/min are normally under supervision of a nephrologist, patients with 15–59 ml/min are generally not and are therefore at increased risk for rDRP. Although we adjusted the eGFR_indexed_ to the actual BSA (eGFR_non-indexed_), the number of patients displaying one or more rDRP (61%) and the median number of rDRP/patient was similar to studies from other medical departments using the eGFR_indexed_ [3, 4]. However, in our study, every second drug was classified as RRD with about 40.0% of the RRD associated with rDRP, which is somewhat higher compared with other studies [3]. One reason for this might be that patients are admitted to an university hospital with more serious health problems, demanding consultation of specialists. This could lead to a higher number of drugs taken by patients at a university hospital compared with non-university hospitals. However, we did not test this hypothesis. Manifest rDRP accounted with 58.5% for the majority of detected rDRP in our study. Use of suboptimal drugs, followed by overdosage and the presence of contraindications, were the causes in most cases.

When distinguishing further in eGFR-subgroups, which represent common dosage frames, more manifest rDRP are seen in lower eGFR-ranges. Of note, more than half of manifest rDRP concerning treatment safety occurred in patients with an eGFR_non-indexed_ 15–29 ml/min. The most frequently prescribed RRD associated with manifest rDRP in our patient population was low molecular weight heparins (LMWH; enoxaparin in this case). The unadjusted dosage of LMWH leads to a higher bleeding risk for patients with RI [30, 33]. Our data indicate that the awareness of correct prescribing of LMWH in patients with RI still seems to be problematic in the ambulant setting and therefore may also be a risk factor in the hospital setting.

Renal function can alter quickly in urologic patients after hospital admission, e.g. impairing in the perioperative period or improving after correction of urinary flow obstructions [34–36]. Therefore, we think it is important not only to focus on manifest rDRP but also to point out potential rDRP that may suddenly become relevant. The leading cause for potential rDRP was overdosage, followed by contraindication or the use of a suboptimal drug. This is in line with the findings of other studies where non-optimal dose and non-optimal drugs were the main causes for rDRP [3, 4]. The most frequently prescribed drug associated with potential rDRP was vitamin D. Indeed, there are still uncertainties regarding the use (indication and type of vitamin D derivative) of vitamin D and its derivatives depending on renal function [7]. Physicians follow recommendations to prescribe vitamin D to patients with RI. As the main activation from colecalciferol to calcitriol takes place in the kidneys, a change of prescription to the activated form has to be considered from a certain stage of kidney disease on. In our experience, this is often neglected and there is a need of further guidance how to prescribe vitamin D derivatives depending on the severity of RI.

Additionally, it has to be kept in mind that some drugs are specifically used against their labeling e.g. hydrochlorothiazide in combination with loop diuretics with an eGFR < 30 ml/min, and should not be classified as rDRP. Thus, it is important to use renal dosing references additionally to the SPC to determine RRD.

In non-hospitalized patients, inappropriate prescribing in RI was found in up to 80% and associated with more ADR, a longer hospital stay and a higher mortality risk [10, 12]. Thus, at hospital admission, screening for affected patients is of great importance as a proactive risk management. Physicians and pharmacists should work together to achieve safer drug prescribing [13]. The most significant reduction of inappropriate prescribing in patients with RI has indeed been observed when physicians received immediate feedback from pharmacists [12]. Prescription review followed by recommendations by a pharmacist has been shown to positively influence clinical outcome and even reduce costs of hospital stay [37]. In our study, for manifest and potential rDRP, the three most often recommended interventions to the physician on ward would have been monitoring, change of dosage and change or discontinuation of a drug.

Furthermore, it is important to recalculate the automatically reported eGFR_indexed_ to the eGFR_non-indexed_ before assessing the medication. In our experience, this recalculation is not usually performed by physicians on wards and the support of a pharmacist would be important. Moreover, the use of medication plans derived from medication reconciliation by a pharmacist at hospital admission, as in this study, can identify patients at risk. It has been shown that these plans are more complete and accurate compared with medication plans prepared by physicians [38, 39].

Some limitations of the study should be considered. This retrospective study focused on rDRP but did not assess possibly related adverse drug reactions, which should be included in future evaluations. Readmissions were included in our study to represent a real-life setting and because patient’s renal function may change over time. However, this might have over- or underestimated the prevalence of RI in our study population. In addition, we assumed comorbidities from the indication of drugs taken by the patients because experiences from previous evaluations revealed that the documentation of diagnoses is often poor. This allows a more complete characterisation of the comorbidities in our study population. However, at the same time, due to drugs given without indication or due to diseases not treated adequately with drugs, errors may be included in our evaluation.

## Conclusion

Our study provides novel evidence that urological patients with RI take a high number of RRD at hospital admission, leading to a substantial number of rDRP. This may be a risk factor for patient safety during the hospital stay. In addition, our analyses demonstrate considerable shifts of patients between eGFR-categories when recalculating eGFR from standard-BSA (indexed eGFR; ml/min/1.73m^2^) to individual BSA (non-indexed eGFR; ml/min) for drug dosing purposes. This is an important point to avoid over- and underdosing or mistakes in contraindications that are frequently overlooked so far.

Future goals should be to develop a risk assessment to simplify the identification of the concerned patients during the pharmacist-led medication reconciliation at admission and to investigate the best way to inform physicians on ward about inappropriate drug use to ensure appropriate prescribing during the hospital stay.

## Electronic supplementary material

ESM 1 (DOCX 88 kb)

## Data Availability

The datasets used and/or analysed during the current study are available from the corresponding author on reasonable request.

## Abbreviations

ADR: adverse drug reactions
AKI: acute kidney injury
BMI: body mass index
BSA: body surface area
CK: creatine kinase
CKD: chronic kidney disease
CKD-EPI: chronic kidney disease epidemiology collaboration
CG: Cockcroft-Gault
eGFR: estimated glomerular filtration rate
rDRP: renal drug-related problems
KDIGO: Kidney Disease Improving Global Outcomes Initiative
LMWH: low molecular weight heparins
RI: renal impairment
RRD: renal risk drugs
SPC: summary of product characteristics
