# Supplementary material for: Correct use of non-indexed eGFR for drug dosing and renal drug-related problems at hospital admission
Source: Eur J Clin Pharmacol. 2020 Jul 10;76(12):1683–93. doi: 10.1007/s00228-020-02953-6 (PMC7661404; doi:10.1007/s00228-020-02953-6)
Supplement: Supplementary file 1 — (DOCX 88 kb) [file 228_2020_2953_MOESM1_ESM.docx]

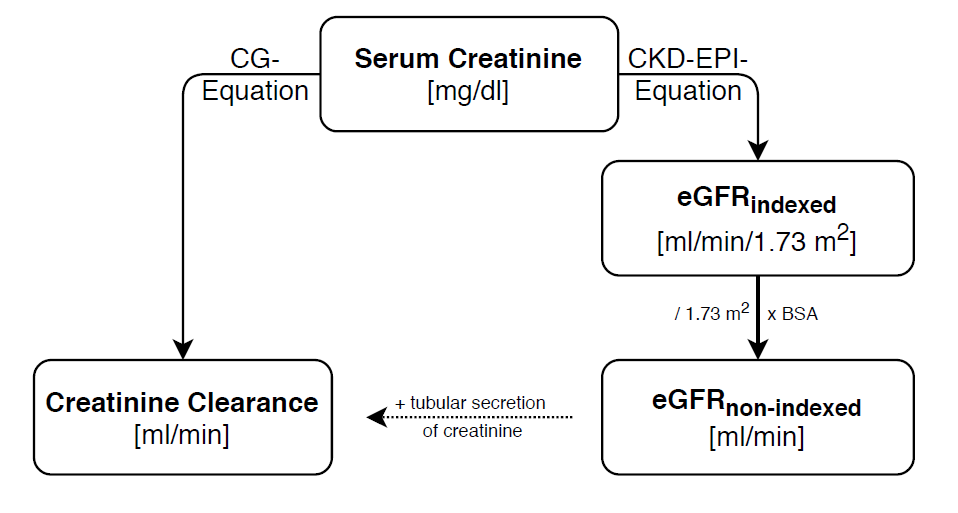


**Figure S1:** Determination of renal function with creatinine-based equations.

**eGFR:** estimated Glomerular Filtration Rate.

**BSA:** Body Surface Area

**CG:** Cockcroft-Gault.

**CKD-EPI:** Chronic Kidney Disease Epidemiology Collaboration.


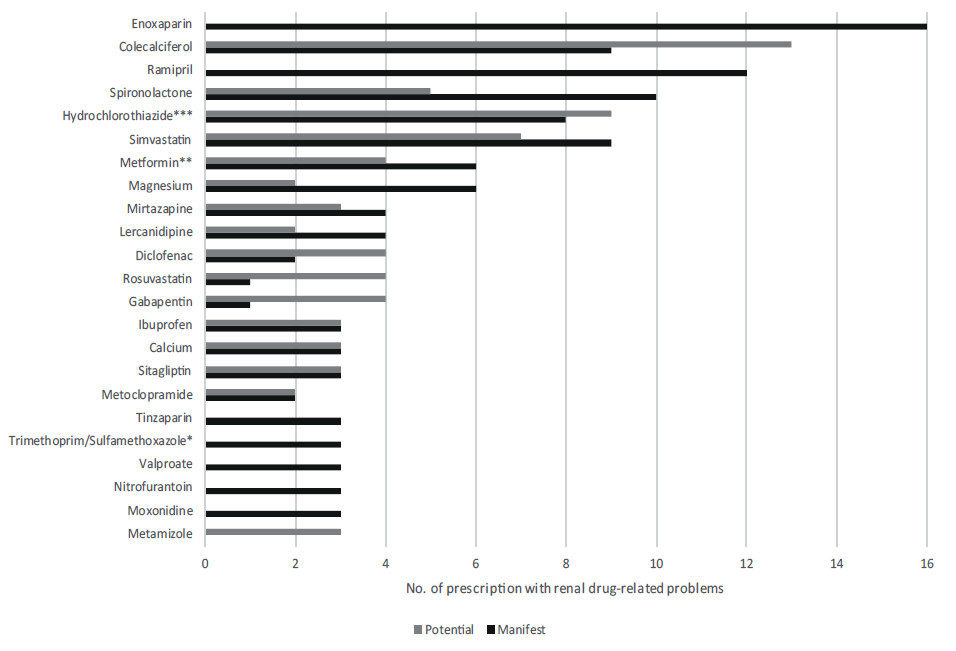


**Figure S2:** Medications most often associated with manifest and potential renal drug-related problem (rDRP) in patients with eGFR_non-indexed_ of 15-59 ml/min and ≥1 drug (n=190).

Potential: eGFR must be monitored, if it decreases, action must be taken.

Manifest: rDRP is currently present.

* Therapeutic dosage, no prophylaxe.

** Metformin contraindicated with eGFR <30 ml/min, dosage adaption with eGFR 30-59 ml/min.

*** HCT as monotherapy contraindicated with eGFR <30 ml/min.
